# Regulation of autophagy by amino acids and MTOR-dependent signal transduction

**DOI:** 10.1007/s00726-014-1765-4

**Published:** 2014-06-01

**Authors:** Alfred J. Meijer, Séverine Lorin, Edward F. Blommaart, Patrice Codogno

**Affiliations:** 1 Department of Medical Biochemistry, Academic Medical Center, University of Amsterdam, Meibergdreef 15, 1105 AZ Amsterdam, The Netherlands; 2 UPRES EA4530, Université Paris-Sud, Faculté de Pharmacie, 5 rue Jean-Baptiste Clément, 92296 Châtenay-Malabry Cedex, France; 3 INSERM U1151-CNRS UMR 8253, Université Paris Descartes, 14 rue Maria Helena Vieira Da Silva CS61431, 75993 Paris Cedex 14, France

**Keywords:** Glutamine, Leucine, Rapamycin, Lysosomes, Mitochondria

## Abstract

Amino acids not only participate in intermediary metabolism but also stimulate insulin-mechanistic target of rapamycin (MTOR)-mediated signal transduction which controls the major metabolic pathways. Among these is the pathway of autophagy which takes care of the degradation of long-lived proteins and of the elimination of damaged or functionally redundant organelles. Proper functioning of this process is essential for cell survival. Dysregulation of autophagy has been implicated in the etiology of several pathologies. The history of the studies on the interrelationship between amino acids, MTOR signaling and autophagy is the subject of this review. The mechanisms responsible for the stimulation of MTOR-mediated signaling, and the inhibition of autophagy, by amino acids have been studied intensively in the past but are still not completely clarified. Recent developments in this field are discussed.

## Introduction

For maintenance of cellular homeostasis, it is not only essential that cell components are synthesized and assembled when required but also that these components are removed and degraded when they are aberrantly synthesized, become damaged or when they are functionally redundant. When these processes are not carried out properly, the cell may either die or turn into a tumor cell in which cell growth proceeds unrestrained.

The major protein degradation systems include the ubiquitin–proteasome pathway (responsible for the quality control of newly synthesized proteins and the degradation of short-lived proteins) (Ciechanover 2012), macroautophagy (responsible for the degradation of long-lived proteins, protein aggregates and entire organelles) (Klionsky and Codogno 2013; Choi et al. 2013; Shen and Mizushima 2014) and chaperone-mediated autophagy (taking care of the removal of specific cytosolic proteins carrying a lysosomal target motif) (Cuervo and Wong 2014). Cross talk between these systems is also possible (Wang et al. 2013).

The process of macroautophagy (hereafter referred to as “autophagy”) has gained tremendous scientific interest in recent years. This is not only because of the partial unraveling of the protein and lipid machinery participating in this complicated cell biological process (Ohsumi 2014; Feng et al. 2014) but also because of the control of autophagic flux by growth factor- and amino acid-dependent signal transduction (Meijer and Codogno 2009; Russell et al. 2014). Above all, however, autophagy gained general interest because dysregulation of the process is implicated in many pathologies. These include, for example, cancer, neurodegeneration, obesity, type 2 diabetes, aging, heart and liver disease, lysosomal storage disorders, bacterial/viral infection and immunity diseases (Rubinsztein et al. 2012; Lavallard et al. 2012; Lieberman et al. 2012; Lorin et al. 2013a; Jiang and Mizushima 2014). In addition, autophagic activity in neurons of the hypothalamus appears to play an essential role in the control of body energy expenditure, appetite and body weight (Kaushik et al. 2011; Lavallard et al. 2012; Quan and Lee 2013). After a brief description of the process of autophagy as we know it today, the focus in this review will be on the regulation of autophagy by amino acids. The history of this fascinating topic, the discovery of amino acid-dependent signaling and possible mechanisms contributing to the inhibition of autophagy by amino acids, with recent developments in this field, will be discussed.

## Autophagy

According to current opinion, the primary function of autophagy is to allow the cell to survive under stress conditions rather than to function as a cell death mechanism (Kroemer and Levine 2008). In the course of autophagy, macromolecules are degraded to small molecule precursors in order to support essential metabolic pathways under these conditions. A classical example at the whole body level is the autophagic production of amino acids, in the liver or elsewhere in the body, for hepatic gluconeogenesis during starvation (Schworer and Mortimore 1979; Ueno et al. 2012), glucose being essential as energy source for brain and erythrocytes under all circumstances.

During autophagy, which occurs in all eukaryotic cells, part of the cytoplasm is surrounded by a double membrane to form an autophagosome that acquires hydrolytic enzymes by fusion with endocytic compartments and lysosomes to form an autophagolysosome. In this process, the outer autophagosomal membrane fuses with the lysosomal membrane, and the inner autophagosomal membrane vesicle is released in the lysosomal lumen ((Meijer and Codogno 2009), for literature) upon which this vesicle, including its sequestered material, becomes degraded. The degradation products (e.g., amino acids) are transported to the cytosol via specific permeases (Mizushima and Klionsky 2007). The rate-limiting step in the entire autophagic pathway is the formation of the autophagosome. This formation starts with the expansion of a membrane core, the isolation membrane, or so-called phagophore (Seglen and Bohley 1992; Klionsky and Seglen 2010). Although progress concerning the origin and the biogenesis of the isolation membrane has been made, many questions still remain to be answered (Lamb et al. 2013; Shibutani and Yoshimori 2014). Very recent data suggest that the phagophore may be built up from the ER-mitochondria contact site (Hamasaki et al. 2013). However, other compartments such as the endoplasmic reticulum-Golgi intermediate compartment (Ge et al. 2013), endosomes and the plasma membrane also contribute to the formation of autophagosomes (Puri et al. 2013). Many different proteins are involved in autophagosome formation. More than 30 proteins have been identified in yeast (Ohsumi 2014; Feng et al. 2014) which are referred to as ATG (Autophagy related) proteins. Seventeen of these proteins are engaged in the biogenesis of the autophagosome, most of which are conserved in mammalian cells (including human cells) but carry different names in order to distinguish them from their yeast counterparts (Stanley et al. 2014). Among these is LC3-I (equivalent to Atg8 in yeast) which, when lipidated with phosphatidylethanolamine (and then named LC3-II), is widely used as a marker of autophagosomes (Klionsky et al. 2012).

Autophagosome formation and degradation can be extremely rapid. For example, in rat hepatocytes in vivo, the autophagic sequestration rate may vary from 0.2 % in the fed state to 1–1.5 % of the cell volume/h in the fasted state (Schworer and Mortimore 1979; Kovacs et al. 1982). In vitro, with isolated hepatocytes or in the perfused liver, in the absence of amino acids, this rate can be as high as 4 %/h (Blommaart et al. 1997a).

In the liver, autophagosomes are synthesized and degraded with a half-life of 8 min (Pfeifer 1977, 1978; Schworer and Mortimore 1979), and such short half-lives also apply to other cell types (Hailey and Lippincott-Schwartz 2009; Shibutani and Yoshimori 2014). Because of this high turnover rate, the steady-state volume of autophagosomes in the cell is low. It is important to stress that the measurement of the steady-state level of autophagosomes (a situation to be compared with the concentration of an intermediate in a metabolic pathway) does not give any information on the magnitude of the autophagic flux as is often assumed, and this notion not only applies to hepatocytes but to other cells as well (Meijer 2009; Klionsky et al. 2012). In order to estimate autophagic flux, at least for in vitro studies with perfused organs or cultured cells, several methods have been recommended. Among these, the most popular are the rate of 3-methyladenine (an inhibitor of autophagosome formation, see below)-sensitive degradation of long-lived proteins and the rate of accumulation of LC3-II (not the level of LC3-II at one time point), which is assumed to represent the rate of autophagosome formation, when lysosomal function and/or fusion is compromised by specific inhibitors (e.g., by chloroquine or bafilomycin, respectively) (Klionsky et al. 2012). The latter method may be complicated by possible feedback interactions caused by the accumulation of autophagosomes (Ktistakis et al. 2011) so that the accumulation of LC3-II may not always be linear with time. Another approach to quantify autophagic flux measures the rate of disappearance of p62/SQSTM1, an adapter protein that serves to carry protein cargo to be degraded to the phagophore (Bjorkoy et al. 2009). However, caution must be taken because in some cell lines amino acids upregulate the transcription of SQSTM1 (Sahani et al. 2014). Other methods, including sophisticated fluorescence techniques, are also available. For a complete description of these techniques, the reader is referred to a recent paper (Klionsky et al. 2012).

**Fig. 1:**
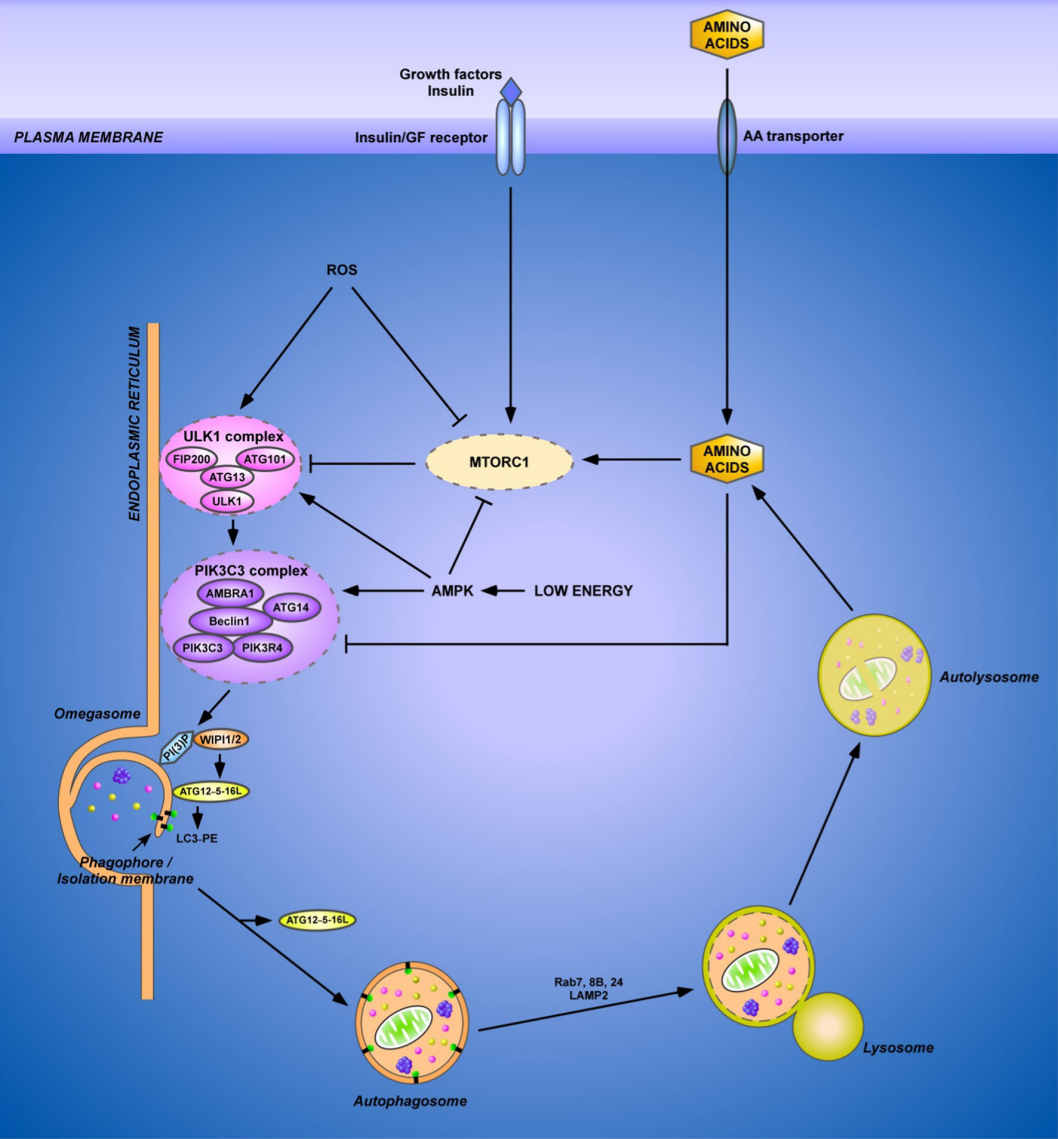
Overview of the autophagic machinery. (Macro)autophagy starts with the nucleation of an isolation membrane, named the phagophore, which surrounds a fraction of the cytoplasm destined for degradation. Upon induction of autophagy, e.g., in starvation, the ULK1 complex localizes to a specialized domain of the ER called the omegasome. This privileged site for the biogenesis of the phagophore forms a cradle where the autophagosomal membrane elongates and acts as a template for the spherical form of the autophagosome. Downstream the ULK1 complex is the PIK3C3 complex, which produces phosphatidylinositol 3-phosphate (PI(3)P) to allow the recruitment of the PI(3)P-binding proteins WIPI1/2 and ZFYVE1/DFCP1. Both contribute to the expansion and the closure of the autophagosome together with the ATG12–ATG5-ATG16L complex and the LC3-phosphatidylethanolamine (LC3-PE) conjugate. Whereas ATG12–ATG5-ATG16L only transiently associates with the autophagosomal membrane, LC3–PE constitutes a specific marker of the autophagosome as it remains associated with the autophagosomal inner membrane. The newly formed autophagosome receives input from the endocytic pathway and ultimately fuses with a lysosome, allowing the degradation of autophagic substrates by lysosomal hydrolases. Fusion of the autophagosome with the lysosome requires the small Rab GTPases (such as Rab7, 8B and 24) and the transmembrane lysosomal protein LAMP2. The products of autophagic degradation, such as amino acids, are recycled to the cytosol where they exert a negative feedback on autophagy initiation. In addition to amino acids, autophagy is also controlled by upstream signaling pathways governed by insulin/growth factors, reactive oxygen species (ROS) and the energy status (through AMPK). Most of these factors regulate the two initiation complexes, ULK1 and PIK3C3. As a master regulator of autophagy, MTORC1 integrates multiple of these upstream signals and controls the activity of the ULK1 complex

**Fig. 2:**
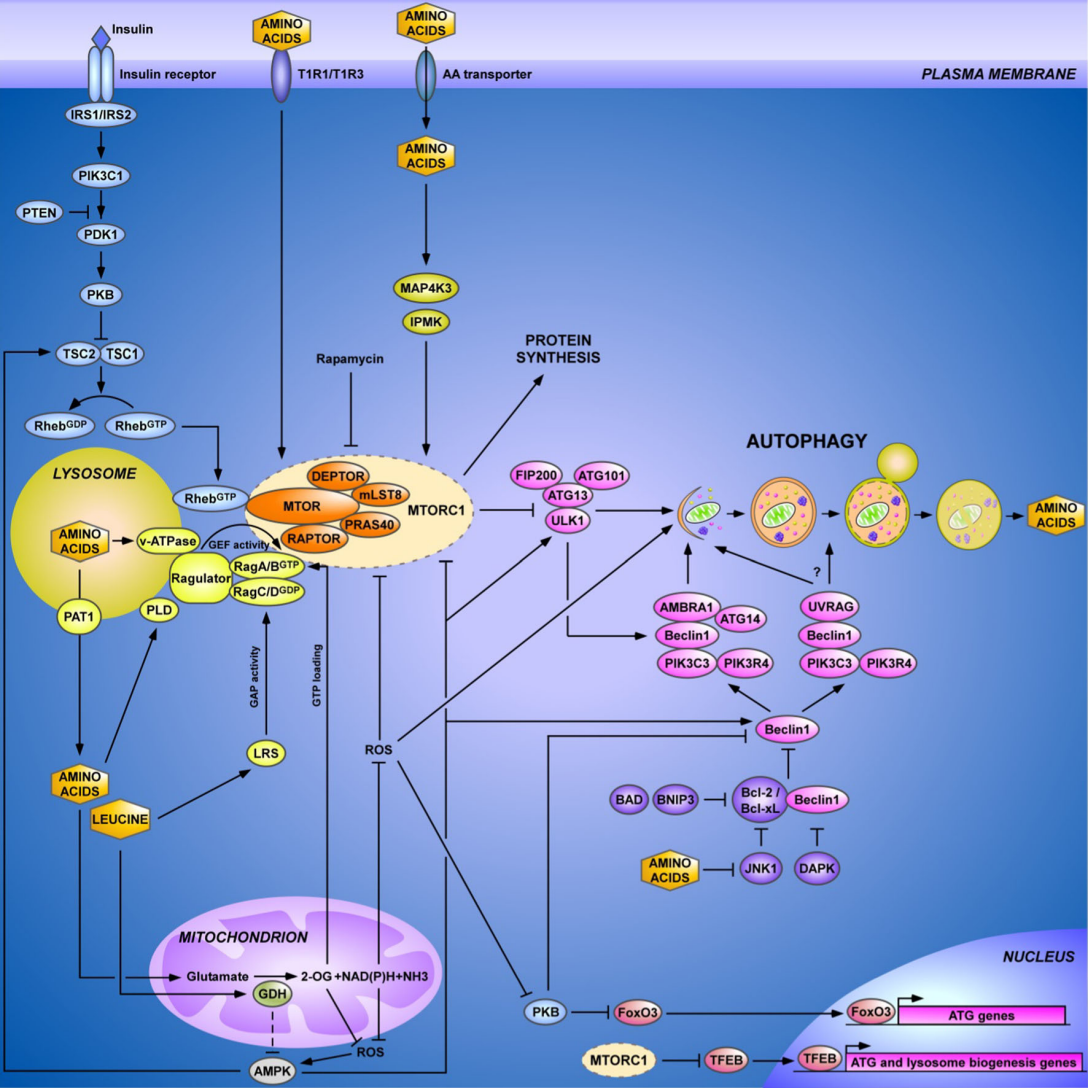
Regulation of autophagy by amino acids. Autophagosome formation is regulated by two major modulators of autophagy, the MTORC1 and PIK3C3 complexes, which integrate amino acid signaling. Under fed conditions, when MTORC1 is fully active, MTORC1 downregulates autophagy by phosphorylating ULK1 and ATG13, which inhibits the ULK1 complex. MTORC1 also inhibits the synthesis of ATG proteins and the synthesis of proteins involved in the biogenesis of lysosomes at the transcription level, by preventing the translocation of TFEB to the nucleus. MTORC1 is activated in two ways: first, by insulin/growth factor signaling which involves PIK3C1, PDK1, PKB and TSC1/TSC2 as signaling components, and second, by amino acids through the Rag GTPases. To be active, MTORC1 has to localize at the lysosomal membrane, where its co-activator Rheb^GTP^ resides. In response to amino acids, Rag promotes the translocation of MTORC1 to the lysosomal membrane and its consecutive activation. Rag proteins are heterodimers of two subunits: RagA/B and RagC/D in which RagA/B is linked to GTP and RagC/D to GDP in the most active form of the dimer. The Rag GTPases are regulated by the v-ATPase, Ragulator and leucyl-tRNA synthetase (LRS). The nucleotide status of RagA/B and of RagC/D is regulated by GATOR and folliculin, respectively (not shown in the figure, for the purpose of clarity; see main text). In response to a rise in the intralysosomal pool of amino acids, the v-ATPase, present in the lysosomal membrane, changes its conformation and recruits Ragulator which displays a guanine nucleotide exchange factor (GEF) activity toward RagA/B. This results in the formation of RagA/B^GTP^ and the activation of MTORC1. In this mechanism, the transporter PAT1, responsible for the efflux of amino acids from the lysosome, controls the concentration of amino acids in the lysosomal lumen and thus the extent of MTORC1 activation. In the presence of cytosolic leucine, binding of leucine to LRS reveals its GTPase-activating protein (GAP) activity toward RagC/D, resulting in the formation of RagC/D^GDP^ and activation of MTORC1. The activity of Rag is also promoted by glutamate dehydrogenase (GDH). This mitochondrial enzyme, which plays a central role in amino acid catabolism, is allosterically activated by leucine. The production of 2-oxoglutarate by GDH may stimulate the loading of RagB with GTP. GDH may also activate MTORC1, and inhibit autophagy, through other distinct mechanisms. (1) The production of NAD(P)H by GDH may lead to the reduction of ROS, a potent activator of autophagy which acts through MTORC1-dependent and MTORC1-independent pathways (i.e., by inhibiting MTORC1 and PKB and by activating AMPK and ATG4). In addition to NAD(P)H, 2-oxoglutarate can also act as a scavenger of ROS, which oxidizes 2-oxoglutarate to succinate non-enzymatically. (2) The production of 2-oxoglutarate by GDH replenishes the citric acid cycle intermediates, increases the rate of ATP production and inhibits AMPK. The fall in AMPK activity may inhibit autophagy by MTORC1-dependent and MTORC1-independent mechanisms (i.e., by inhibition of TSC1/TSC2, stimulation of MTORC1 and inhibition of the ULK1 complex and of Beclin1). Probably acting in parallel with the Rag GTPase pathway, MAP4K3 and IPMK are other proteins involved in the regulation of MTORC1 by amino acids. The extracellular pool of amino acids may be sensed by the plasma membrane amino acid receptor T1R1/T1R3, which regulates MTORC1 and autophagy. The other major protein complexes controlling autophagy contain the Beclin1 protein. The core proteins of these two complexes are Beclin1, PIK3C3 and PIK3R4. When associated with ATG14 and AMBRA1, Beclin1 stimulates the early steps of autophagosome formation, downstream of the ULK1 complex. When associated with UVRAG, Beclin1 is mainly involved in the formation and maturation of autophagosomes. In response to amino acids, the protein kinase JNK1 becomes inhibited, leading to the formation of a stable complex between Beclin1 and Bcl-2 which sequesters Beclin1 and results in inhibition of autophagy. Beclin1 is also inhibited by PKB-dependent phosphorylation, which likewise inhibits autophagy. Long-term regulation of autophagy by PKB occurs by phosphorylation of FoxO3, another transcription factor (in addition to TFEB) responsible for the synthesis of ATG proteins. For further details, see main text. For reasons of clarity, the control of the inhibitory acetylation of ATG proteins by mitochondrial amino acid catabolism, which increases the concentration of acetyl CoA in the cytosol, is not indicated in the figure

Autophagy is initiated by activation of a protein complex containing the protein kinases ULK1 and 2 (the mammalian counterpart of yeast Atg1), the proteins ATG13, FIP200 (yeast Atg17), ATG101 and many other components (Wong et al. 2013; Russell et al. 2014). Phosphorylation of ATG13 by ULK1/2 promotes the association of these proteins and is essential for initiation of autophagy (Fig. 1). ULK1 binds to membranes through its C-terminal domain (Chan et al. 2009). Downstream of the ULK1/2 complex is a lipid kinase, PIK3C3 (yeast Vps34), which produces PI(3)P and which is part of another protein complex also containing the regulatory protein PIK3R4 (yeast Vps15), the proteins Beclin1 (yeast Atg6/Vps30), ATG14 (also known as BARKOR). In the Beclin1-PIK3C3 core complex, Beclin1 can interact with several proteins such as AMBRA1, UVRAG and VMP1 to control autophagosome formation and/or maturation (Wirth et al. 2013) (cf. also Fig. 2). ULK1 phosphorylates both AMBRA1 and Beclin1 to initiate autophagosome formation (Russell et al. 2013; Lorin et al. 2013a). This results in activation of PIK3C3. Production of PI(3)P recruits PI(3)P-binding proteins involved in the initial formation of the autophagosome, WIPI1/2 (yeast Atg18), ZFYVE1/DFCP1 and two ubiquitin-like conjugation systems ATG12–ATG5-ATG16L and LC3-phosphatidylethanolamine (LC3-II) (Polson et al. 2010; Weidberg et al. 2011; McAlpine et al. 2013). PI(3)P is also required to join the ends of the autophagosomal membrane in statu nascendi (termed the “omegasome” because of its cup-formed shape) (Axe et al. 2008). The final fusion of the newly formed autophagosome with the lysosome requires small Rab GTPases, such as Rab7, Rab8 and Rab24, and the transmembrane protein LAMP2 (Simonsen and Tooze 2009; Ao et al. 2014).

Association of Beclin1 with PIK3C3 is an essential step in autophagosome formation. However, Beclin1 is a BH3 (Bcl-2 homology domain) protein and can also associate with the anti-apoptotic proteins Bcl-2/Bcl-xL which contain a BH3-binding groove. This means that association of Beclin1 with these anti-apoptotic proteins is inhibitory for autophagy and their dissociation is essential to allow Beclin1 to bind to PIK3C3 and to initiate autophagy. The dissociation of the inhibitory Beclin1–Bcl-2 complex is promoted by either JNK-1-mediated phosphorylation of Bcl-2, by phosphorylation of Beclin1 mediated by the tumor suppressor DAPK (a Ca^2+^-calmodulin-activated protein kinase), or by displacement of Beclin1 from its complex with Bcl-2 by other BH3-containing proteins such as the proapoptotic protein BAD and the proautophagic protein BNIP3 ((Meijer and Codogno 2009; Mariño et al. 2014a), for literature) (Fig. 2).

## The insulin-amino acid-MTOR signaling pathway

The initiation of autophagosome formation is controlled by the insulin/growth factor-PI3KC1-PKB-TSC-MTOR-mediated signal-transduction pathway, which integrates hormonal, nutritional, cellular energy and oxidative stress inputs and which controls all major metabolic pathways (Meijer and Codogno 2009; Avruch et al. 2009; Kim and Guan 2011; Laplante and Sabatini 2012; Shanware et al. 2013; Cornu et al. 2013; Kim et al. 2013b).

The initial part of this signaling pathway, upstream of MTOR, an evolutionary conserved serine/threonine protein kinase which was first described by (Heitman et al. 1991), involves the insulin receptor, IRS1 and IRS2, PIK3C1, producing PI(3,4,5)P3 and PI(3,4)P2, PDK1 and PKB (Fig. 2). For activation of MTOR, the presence of insulin alone is not sufficient: The presence of amino acids is indispensable (Blommaart et al. 1995; Hara et al. 1998; Meijer and Codogno 2009; Barazzoni et al. 2012) (see below).

The second part of the insulin-signaling pathway, downstream of MTOR, may involve components such as S6K, 4E-BP1, eIF2α kinase and eEF2 kinase. Phosphorylation of these proteins by MTOR promotes protein synthesis.

MTOR itself is part of two multiprotein complexes. MTORC1 contains MTOR, RAPTOR, mLST8, and the inhibitory proteins DEPTOR and PRAS40. MTORC2 (not indicated in Fig. 2) contains MTOR, RICTOR, mLST8, mSin1, PROTOR and DEPTOR. Activation of MTORC1 simultaneously inhibits autophagy and stimulates protein synthesis (Meijer and Codogno 2009; Laplante and Sabatini 2012). Activation of MTORC2 does not have these effects but rather phosphorylates, and activates, PKB. In liver, for example, this results in stimulation of glycolysis and lipid biosynthesis (Hagiwara et al. 2012). MTORC1 and MTORC2 can be distinguished on the basis of their sensitivity toward inhibition by rapamycin which only inhibits MTORC1 (Sarbassov et al. 2006). Although the kinase activity of MTOR in MTORC2 is insensitive to acute rapamycin treatment, chronic exposure to the drug can disrupt its structure and results in insulin resistance (Sarbassov et al. 2006; Ye et al. 2012).

The activity of MTOR in MTORC1 is inhibited by the heterodimer TSC1/TSC2: it acts as a GTPase-activating protein complex for the small G-protein Rheb. Rheb^GTP^, not Rheb^GDP^, binds and activates MTOR. PKB phosphorylates TSC2, which inactivates the TSC1/TSC2 complex and stimulates MTORC1 (Fig. 2). In addition, MTORC1 is stimulated by PKB-dependent phosphorylation of PRAS40 (Manning and Cantley 2007).

## Interaction of the insulin-amino acid-MTOR signaling pathway with autophagy

The insulin/growth factor-PIK3C1-PKB-MTOR signaling pathway inhibits autophagosome formation at several levels.

MTOR phosphorylates ULK1/2 and ATG13 which results in their dissociation and in inactivation of ULK (Ganley et al. 2009; Jung et al. 2009; Mizushima and Komatsu 2011; Lorin et al. 2013a). MTOR also inactivates PIK3C3, albeit indirectly, i.e., when the lipid kinase is associated with ATG14, by phosphorylating ATG14 (Yuan et al. 2013). Long-term regulation of autophagy by MTOR occurs by phosphorylation of the transcription factor EB (TFEB), a master regulator of the synthesis of ATG proteins and of the biogenesis of lysosomes. This phosphorylation prevents translocation of TFEB to the nucleus (Settembre et al. 2012). In addition, the activity of TFEB is inhibited by nutrient- and growth factor-dependent extracellular signal-regulated kinase 2 in an MTOR-independent fashion (Settembre et al. 2011) (Fig. 2). The same protein kinase can also inhibit autophagy in the short term by phosphorylation of TSC1/TSC2 (Kim et al. 2013b).

The complexity of the regulation of autophagy by MTOR is illustrated by the fact that MTOR not only inhibits ULK1 but that, conversely, ULK1 also inhibits MTOR by phosphorylation. This inhibition of MTORC1 by ULK1 may serve to amplify and stabilize initially small changes in nutrient signaling (Chang et al. 2009; Jung et al. 2011).

Autophagy is also controlled by PKB. Short-term regulation occurs by PKB-dependent phosphorylation of Beclin1 (Wang et al. 2012) (Fig. 2). Long-term regulation by PKB occurs by phosphorylation of FoxO3, another transcription factor responsible for the synthesis of ATG proteins (Mammucari et al. 2007). This set of ATG proteins differs from that controlled by TFEB (Settembre et al. 2011).

In addition to these various phosphorylation mechanisms, PI(3,4,5)P3 and PI(3,4)P2, the products of PIK3C1, are inhibitors of autophagy, possibly because these lipids compete with PI(3)P (Petiot et al. 2000; Meijer and Codogno 2009; Farrell et al. 2013).

## Regulation of autophagy by amino acids and hormones: some history

Initial studies on the regulation of autophagy were primarily carried out with liver, both in vitro with the perfused liver and isolated hepatocytes, and in vivo. The process was known to be product inhibited by amino acids (Schworer and Mortimore 1979; Seglen et al. 1980), stimulated by glucagon (Ashford and Porter 1962; Deter et al. 1967) and inhibited by insulin (Mortimore and Mondon 1970), but the mechanisms underlying these effects were unknown at the time. Interestingly, in the perfused liver insulin and glucagon did only affect autophagy at intermediate amino acid concentrations but had no effect in the absence of amino acids when autophagic flux was maximal, nor did they have an effect in the presence of high concentrations of amino acids that inhibited autophagy maximally (Schworer and Mortimore 1979; Mortimore et al. 1987). It was also clear that regulation of autophagy by these factors was primarily exerted at the level of autophagosome formation (the autophagic sequestration step), although effects at later stages in the autophagic pathway could not be excluded (Hoyvik et al. 1991; Luiken et al. 1996). One confounding factor in experiments in vitro has been the accumulation of ammonia, derived from amino acid degradation which was found to increase the intralysosomal pH because of its acidotropic properties (Seglen 1977; Kadowaki and Kanazawa 2003). Even today, this complication is not always appreciated and unawareness of it may lead to erroneous conclusions (Meijer 2009).

Our own interest in the regulation of autophagy by amino acids was triggered by experiments performed with freshly isolated, perifused, rat hepatocytes under true steady-state conditions. Intriguingly, we found that a simple combination of leucine and alanine (which was selected on the basis of existing literature) at near-physiological concentrations could mimic the inhibitory effect on autophagic proteolysis of a complete mixture of all amino acids; either alanine or leucine alone had no effect (Leverve et al. 1987). Similar observations were made by Mortimore and colleagues for the perfused liver (Mortimore et al. 1988). Combinations of low concentrations of leucine and either proline, glutamine, or asparagine were also effective as inhibitors of autophagy (Caro et al. 1989). With leucine present, the inhibition by alanine, but not by proline, glutamine, or asparagine, was sensitive to inhibition by amino-oxyacetate, a transaminase inhibitor (Leverve et al. 1987; Caro et al. 1989). This indicated that metabolism of alanine, but not that of leucine, was required for its inhibitory effect on autophagy. As conversion of proline and glutamine to glutamate and of asparagine to aspartate does not require transamination reactions, we suspected that a combination of leucine with intracellular glutamate or aspartate (which does not readily leave hepatocytes) was sufficient to inhibit autophagy maximally. Indeed, in the presence of leucine and either alanine, proline, glutamine, or asparagine, there was an inverse relationship between the rate of autophagic proteolysis and the intracellular concentration of glutamate plus aspartate (Caro et al. 1989). However, the underlying mechanism for the inhibition of autophagy was entirely unknown. Interestingly, the same amino acids that inhibited hepatic autophagic proteolysis were also known to inhibit proteolysis in other tissues, including skeletal muscle, heart and kidney, with leucine being most effective (Blommaart et al. 1997a).

A new factor in the control of autophagic protein degradation was introduced with the observation, initially reported for liver but later extended to other cell types, that an increase in cell volume was able to mimic several of the anabolic properties of insulin, including inhibition of glycogenolysis (Lang et al. 1989), inhibition of proteolysis (Hallbrucker et al. 1991), stimulation of protein synthesis (Stoll et al. 1992) and lipogenesis (Baquet et al. 1991). Cell swelling was induced by either hypo-osmotic conditions or by Na^+^-dependent concentrative transport of amino acids across the plasma membrane. We suspected that intracellular accumulation of impermeant metabolites such as glutamate and aspartate, derived from amino acid degradation, also helped to increase the intracellular osmolarity and in this way contributed to cell swelling. In freshly isolated hepatocytes, cell swelling was found to mimic the effect of insulin in that it potentiated the ability of a complete mixture of amino acids, or of a combination of leucine with tyrosine and phenylalanine, to inhibit autophagic proteolysis (Meijer et al. 1993; Luiken et al. 1994).

Around the same time, our group in Amsterdam, in collaboration with the group of Hue in Brussels, studied the mechanism of the stimulation by amino acids of glycogen synthesis from glucose. This stimulation, first reported by Katz and colleagues (Katz et al. 1976), had been investigated by many laboratories but its mechanism was never elucidated. We discovered that the synthesis of glycogen, too, was due to an amino acid-induced increase in cell volume (Baquet et al. 1990). Leucine, which did not increase cell volume, had no effect, indicating that the mechanism of the stimulation of glycogen synthesis by amino acids differed from that of their inhibition of autophagy. It was known at the time that, in response to an increase in cell volume, cells undergo “regulatory volume decrease” in which they try to restore, at least in part, the original volume by releasing KCl (Hoffmann and Simonsen 1989; Häussinger 1996). We then found that the enzyme glycogen synthase phosphatase was inhibited by intracellular chloride and that the fall in chloride in response to cell swelling was sufficient to activate this enzyme and thus the synthesis of glycogen (Meijer et al. 1992). With regard to autophagy, the mechanism of inhibition by cell swelling was, and still is, not clear. Perhaps chloride ions are involved because they accompany protons, driven by the ATP-dependent lysosomal proton pump, to the lysosomal interior (Ishida et al. 2013). Very recent evidence suggests that this may, indeed, be the case (Hosogi et al. 2014). An effect of cell swelling on the cytoskeleton, mediated by integrins, is another, or additional, possibility (Häussinger et al. 2006).

In summary, in the control of autophagy, leucine appeared to be the most inhibitory amino acid. In addition, its anti-proteolytic effect was potentiated by insulin and by cell swelling.

## Discovery of amino acid signaling and its relationship with autophagy

Early studies by Seglen and coworkers with protein kinase and protein phosphatase inhibitors indicated that protein phosphorylation was involved in the regulation of autophagy in hepatocytes, but the connection with amino acids was not made (Holen et al. 1992, 1993).

A breakthrough in our understanding of the mechanism by which amino acids inhibit autophagy (and stimulate protein synthesis) was obtained by the Amsterdam group in a study carried out with [^32^P]P_i_-labeled rat hepatocytes. It was discovered that the same amino acid mixtures that inhibited autophagy greatly stimulated (up to fivefold) the phosphorylation of a protein that was identified as ribosomal protein S6, with rapid kinetics (*t*_1/2_, 10 min). The stimulation of S6 phosphorylation by amino acids was not due to the inhibition of a protein phosphatase acting on S6 (Luiken et al. 1994; Blommaart et al. 1995). Dephosphorylation of S6 followed similar rapid kinetics upon withdrawal of amino acids or by addition of rapamycin. We observed synergy between either insulin or cell swelling and low concentrations of a complete mixture of amino acids, not only with regard to their inhibition of autophagic proteolysis but also with regard to their ability to promote S6 phosphorylation. Addition of insulin alone, in the absence of added amino acids, did not stimulate S6 phosphorylation. In the presence of high concentrations of amino acids, S6 phosphorylation was maximal and not further increased by insulin. Glucagon, on the other hand, stimulated autophagy and inhibited S6 phosphorylation at low, but not at high concentrations of amino acids or in their absence. Among various amino acids, leucine, not valine, appeared to be very effective. Under several incubation conditions, with different amino acid mixtures, in the absence or presence of insulin and glucagon, there appeared to be a linear relationship between the percentage of inhibition of autophagic proteolysis (measured in the presence of a low concentration cycloheximide sufficient to inhibit simultaneous protein synthesis) and the degree of phosphorylation of S6 (Blommaart et al. 1995). Amino acid-induced S6 phosphorylation was completely prevented by rapamycin, indicating that MTOR and S6K were components of the signaling pathway. Of great significance was the fact that rapamycin could partly (albeit not completely, this will be explained later) reverse the inhibition of autophagy by amino acids. It should be stressed, however, that in the absence of cycloheximide, rapamycin also did not completely inhibit protein synthesis. Because at that time MTOR signaling was known to be involved in the regulation of protein synthesis, it was concluded that (global) protein synthesis and (autophagic) protein degradation were under the opposite control by the same signaling pathway, which was considered to be metabolically efficient (Blommaart et al. 1995).

These studies were the first to demonstrate that amino acids were essential for MTOR-mediated signaling and that this signaling was connected to the regulation of autophagy. They were also the first demonstration that rapamycin was able to stimulate autophagy.

The stimulation of autophagy by rapamycin was confirmed several years later for other cell types (Mordier et al. 2000; Eskelinen et al. 2002; Moazed and Desautels 2002), including yeast (Noda and Ohsumi 1998), and rapamycin is nowadays widely used as an activator of autophagy (Rubinsztein et al. 2012). Torin1, a newly developed ATP-competitive inhibitor of MTOR, acts similarly (Thoreen et al. 2009).

The ability of amino acids to stimulate signaling even in the absence of insulin, and the synergy of amino acids with insulin, was confirmed for hepatocytes (Krause et al. 1996) and for other insulin-sensitive cell types, including muscle cells, adipocytes, hepatoma cells, CHO cells and pancreatic β-cells (Hara et al. 1998; Wang et al. 1998; Fox et al. 1998; Patti et al. 1998; Xu et al. 1998; Iiboshi et al. 1999). In these studies, it was shown that, in addition to S6, other downstream targets of MTOR such as S6K, 4E-BP1, eIF2α kinase (the equivalent of Gcn2 in yeast) and eEF2 kinase were found to be phosphorylated in response to amino acids. However, these studies also indicated that amino acids did not affect PKB activity and PIK3C1, signaling proteins upstream of MTOR that were known to be stimulated by insulin. It was also demonstrated that in case insulin alone, in the absence of added amino acids, stimulated MTOR signaling, this was dependent on the presence of amino acids that were generated by autophagic proteolysis (Shigemitsu et al. 1999a; Beugnet et al. 2003; Duran et al. 2011). Noteworthy is that the kinetics of the stimulation by amino acids of MTOR-mediated phosphorylations in these various cell types were similar to those observed by Blommaart et al. (1995) with regard to S6 phosphorylation in hepatocytes. They were also similar to the rapid phosphorylations reported very recently in an extensive study on the early temporal dynamics of the phosphoproteome in breast cancer cells upon initiation of autophagy by either withdrawal of amino acids or by addition of rapamycin (Rigbolt et al. 2014).

Most studies agreed that leucine (but not the other branched-chain amino acids), independent of the cell type, was the most effective amino acid in stimulating signaling but that, in addition, some other amino acids were required (Hara et al. 1998; Wang et al. 1998; Fox et al. 1998; Patti et al. 1998; Xu et al. 1998; Kimball et al. 1999; Shigemitsu et al. 1999b; van Sluijters et al. 2000; Lynch et al. 2000; Atherton et al. 2010). In analogy with the inhibition of autophagy and the stimulation of S6 phosphorylation by amino acids (see above), it was proposed that leucine, in combination with amino acid-induced cell swelling, would be sufficient to stimulate signaling (van Sluijters et al. 2000). In line with this, Krause et al. (2002b) showed synergy between glutamine, a potent amino acid in promoting cell swelling, and leucine with regard to S6K phosphorylation in hepatocytes. In intestinal cells, with their rapid growth, in addition to glutamine and leucine, arginine has also been mentioned as an activator of MTOR signaling (Nakajo et al. 2005; Marc and Wu 2009). In CHO cells, arginine also stimulated signaling albeit less effective than leucine (Hara et al. 1998). Although never considered, it is possible that the effect of arginine may be attributed, at least in part, to glutamate produced from arginine by the combined actions of arginase, ornithine aminotransferase and pyrroline 5-carboxylate dehydrogenase. Involvement of NO production from arginine can also not be excluded (Angcajas et al. 2014).

In order to account for the synergy between amino acids and insulin with respect to the activation of MTOR, it had to be assumed that MTOR received two parallel input signals, one from the insulin-PIK3C1-PKB signaling branch and one from amino acids (cf. (van Sluijters et al. 2000; Shah et al. 2000), for reviews). We now know that this is, indeed, the case (cf. Fig. 2): Insulin activates MTOR through PKB-dependent phosphorylation of TSC2, which results in activation of Rheb, and of PRAS40 as discussed earlier, while amino acids separately activate MTOR by mechanisms to be discussed below. However, the relative contribution of insulin and amino acids in the regulation of MTORC1 and autophagy may be tissue dependent. Thus, in mice in vivo, insulin appeared to be more potent in stimulating MTORC1 and inhibiting autophagy in muscle than in liver. By contrast, amino acids were more potent in the control of MTORC1 and autophagy in the liver than in muscle (Naito et al. 2013). This difference between the two tissues was tentatively ascribed to the regulation of MTORC1 and of autophagy by glucagon in the liver, but not in muscle. Another possibility is that there are differences in the intramuscular and intrahepatic amino acid concentrations.

S6K was also shown to phosphorylate, and inhibit, IRS1 (Tremblay and Marette 2001). Although this feedback effect of S6K has been proposed to participate in the etiology of insulin resistance (Um et al. 2004; Efeyan et al. 2014), it may also act as a mechanism to prevent overactivation of the MTOR pathway in order to allow some autophagy to continue even under nutrient-rich conditions (Meijer and Codogno 2009).

The inhibition of MTOR signaling by glucagon was later shown to proceed through a mechanism dependent on protein kinase A (Kimball et al. 2004), which stimulates autophagy (Mavrakis et al. 2006). It must be pointed out that, in contrast to mammalian cells, activation of protein kinase A in yeast cells inhibits autophagy (Stephan et al. 2009). In this context, it is important to stress that the function of cAMP in yeast, in contrast to that in mammalian cells, is to stimulate cell growth (Thevelein and de Winde 1999).

## The requirement of PI(3)P for autophagy

Although in the presence of amino acids, rapamycin was able to stimulate autophagy, interruption of signaling by the PIK3C1 inhibitors wortmannin and LY294002, unexpectedly, did not. Even more surprising was the finding that in the absence of amino acids, with maximal autophagic flux, these two compounds strongly inhibited autophagy (Blommaart et al. 1997b). At the time, mammalian cells were known to contain both PIK3C1 and PIK3C3 while yeast only contained the homolog of PIK3C3, Vps34 (and its adapter protein Vps15). Vps34 was known to be involved in processes requiring membrane flow (Schu et al. 1993), but its relationship with autophagy was not yet studied in this organism. Because the phosphatidylinositol 3-kinase inhibitors wortmannin and LY294002 were not specific in that they could not distinguish between PIK3C1 and PIK3C3, it was postulated that perhaps PIK3C3, and thus its product PI(3)P, might be essential for autophagy (Blommaart et al. 1997a, b). This hypothesis was tested, and confirmed, in transfection experiments using HT-29 cells, a human colon cancer cell line, which showed that PI(3)P was, indeed, required for autophagy. Moreover, the products of PIK3C1, PI(3,4)P2 and PI(3,4,5)P3 acted as inhibitors of autophagy (Petiot et al. 2000). In further support of this, it was found that overexpression of PTEN, which removes the phosphate from the 3-position of PI(3,4)P2 and PI(3,4,5)P3, increased autophagy (Arico et al. 2001).

These observations were the first to show that PI(3)P participated in the process of autophagy. They have been amply confirmed for both mammalian cells and yeast (Kihara et al. 2001; Suzuki et al. 2001; Jaber et al. 2012; Schink et al. 2013; Cheng et al. 2014).

## 3-Methyladenine

An important spin-off from these studies was our finding that 3-methyladenine, a specific inhibitor of autophagy described by Gordon and Seglen in as early as 1982 (Seglen and Gordon 1982), and which has proven to be extremely useful in studies on autophagy (to this day), turned out to be an inhibitor of PIK3C3. This provided a satisfactory explanation for its mechanism of action (Blommaart et al. 1997b; Petiot et al. 2000).

## Autophagy regulation and energy: the role of AMPK

Apart from being activated by insulin and amino acids, MTOR was also found to be affected by the cellular energy state. Initial studies with HEK293 cells indicated that MTOR may also act as a sensor of the intracellular ATP concentration (Dennis et al. 2001). It was noted that among various protein kinases the *K*_m_ of MTOR for ATP in vitro was exceptionally high and within the physiological (mM) range of ATP concentrations. Because intracellular AMP is much more sensitive than ATP as an indicator of the cellular energy state, being connected to ATP in the reversible adenylate kinase reaction, AMPK was later considered as another candidate controlling MTOR activity. Indeed, studies simultaneously reported by several laboratories, including our own, demonstrated that, rather than the decreased ATP concentration, it was the activation of AMPK that was responsible for the inhibition of MTOR when energy falls short (Fig. 1) (Dubbelhuis and Meijer 2002; Bolster et al. 2002; Larsen et al. 2002; Krause et al. 2002a; Kimura et al. 2003). In agreement with the notion that inhibition of MTOR results in stimulation of autophagy (as discussed earlier), and also in line with its function to stimulate catabolism (Hardie 2007), it was reported, first by us (Meley et al. 2006) and later by others (Hoyer-Hansen et al. 2007; Liang et al. 2007), that AMPK is essential for autophagy. The activation of AMPK also underlied the stimulation of autophagy in cerebral (Adhami et al. 2006) and cardiac ischemia (Matsui et al. 2007).

The mechanism by which AMPK inhibited MTOR was twofold. The first mechanism proceeded via AMPK-mediated phosphorylation, and activation, of TSC2 (Inoki et al. 2003; Corradetti et al. 2004) which catalyzes the conversion of Rheb^GTP^ to Rheb^GDP^. The second mechanism proceeded via phosphorylation, and inactivation, of RAPTOR (Gwinn et al. 2008) (Fig. 2).

The stimulation of autophagy by AMPK, however, was not only through inhibition of MTOR but also occurred by AMPK-mediated phosphorylation, and activation, of ULK1 (Egan et al. 2011; Kim et al. 2011a). Interestingly, ULK1 is thus phosphorylated by both MTOR and AMPK, but at different sites, with opposing effects on ULK1 activity (Meijer and Codogno 2011). In addition, it was recently shown that in response to glucose starvation AMPK phosphorylates, and activates, Beclin1 provided it is associated with PIK3C3 and ATG14 (Kim et al. 2013a). Thus, as ULK1, Beclin1 is phosphorylated at different sites, with opposing effects: in this case by AMPK (activation) and, as discussed earlier, by PKB (inhibitory) (Fig. 2).

Inhibition of AMPK activity by amino acids (cf. next paragraph), and thus inhibition of Beclin1, would account for an earlier observation showing that amino acids decreased Beclin1-associated PIK3C3 activity (Tassa et al. 2003). An additional explanation is that amino acids promote the association between Beclin1 and Bcl-2, and thus Beclin1 sequestration (Pattingre et al. 2005), through inhibition of JNK1-mediated phosphorylation of Bcl-2 (Wei et al. 2008).

Yet another mechanism by which AMPK can promote autophagy may proceed through activation of Sirtuin1, an NAD-dependent protein deacetylase (Canto et al. 2009; Ruderman et al. 2010). Sirtuin1, which increases in starvation, is known to stimulate autophagy by increasing the deacetylation of several ATG proteins (Lee et al. 2008). In addition, Sirtuin1 stimulates the deacetylation of FoxO3 which also results in activation (Canto et al. 2009). Conversely, acetylation of ATG proteins, which inhibits their activity, is brought about by the acetyltransferase P300 (also known as EP300) (Lee and Finkel 2009).

In principle, the inhibition of AMPK by amino acids would be an attractive mechanism to account for their ability to inhibit autophagy and to stimulate MTOR activity. Such an effect of amino acids on AMPK could be mediated by glutamate dehydrogenase, which plays a central role in amino acid catabolism and which is known to be specifically stimulated by leucine, not by the other branched-chain amino acids (Sener and Malaisse 1980; Fahien et al. 1990). This enzyme produces 2-oxoglutarate, replenishes citric acid cycle intermediates and thus helps to increase the rate of ATP production. Indeed, in many studies, inhibition of AMPK by amino acids has been observed (Xiao et al. 2011; Ghislat et al. 2012; Li et al. 2013). This was not always the case, however (Krause et al. 2002a; Kim et al. 2011a; Wauson et al. 2012; Duran et al. 2013; Rahman et al. 2014).

Because metabolism differs among cell types, e.g., with differences in the use of oxidizable substrates, it is understandable that the effect of amino acids on AMPK may vary. Although it is possible that AMPK is involved in amino acid regulation of autophagy and of MTOR signaling in some cell types, other amino acid sensing mechanisms may also exist. These will be discussed below.

## Mechanisms of amino acid signaling

The mechanism by which amino acids stimulate MTOR activity, and inhibit autophagy, has remained, and still is to some extent, an enigma for a long time. Amino acids do not directly stimulate signaling upstream of MTOR, as discussed above, nor do they stimulate MTOR directly (van Sluijters et al. 2000; Kim and Guan 2011; Laplante and Sabatini 2012). However, as discussed in the previous paragraph, they can do so indirectly by inhibition of AMPK, depending on the cell type. As also mentioned earlier, in most cell types, among the various amino acids, leucine, but not the other branched-chain amino acids valine and isoleucine, is most potent in inhibiting autophagy and stimulating MTOR, and non-metabolizable analogs of leucine could mimic its effect. Catabolism of leucine does not seem to be required (Lynch et al. 2003). Indeed, fibroblasts from patients with defects in leucine catabolism displayed enhanced MTOR activity (Schriever et al. 2013). However, a role for leucine catabolites in MTOR signaling cannot entirely be ruled out. Thus, in experiments in man, administration of the leucine metabolite β-hydroxy-β-methylbutyrate, a compound that in clinical settings is used to treat disease-related muscle wasting, has been shown to stimulate skeletal muscle growth through the MTOR pathway (Wilkinson et al. 2013). It cannot be excluded that this compound, by serving as an oxidizable substrate, decreases AMPK activity and in this way stimulates MTOR signaling. A link between β-hydroxy-β-methylbutyrate-stimulated MTOR activity and the control of autophagy was not established.

It is concluded that any mechanism of amino acid sensing must account for the high leucine specificity.

Several factors involved in amino acid-MTOR signaling have been described in the past and have been reviewed in detail elsewhere (Dann and Thomas 2006; Avruch et al. 2009; Meijer and Codogno 2009; Kim and Guan 2011; Laplante and Sabatini 2012) (Fig. 2). These include, a.o., MAP4K3, PIK3C3, the G-protein Rheb, proton-assisted amino acid transporters in the lysosomal membrane, the Rag GTPases, leucyl-tRNA synthetase, the adapter protein p62, phospholipase D and inositol polyphosphate multikinase. The evidence in support of these various factors relied on the observation that elimination of any one of them strongly interfered with the ability of amino acids to stimulate MTOR signaling. In none of these studies, however, the nature of the primary amino acid sensor was identified. As will become clear, some of these factors are part of one mechanism responsible for the activation of MTOR activity by amino acids, whereas others participate in mechanisms that are independent of, but act in parallel to, each other.

## Rheb

A potential mechanism of amino acid sensing was suggested by the observation that amino acids, leucine in particular, promote the association of Rheb with MTOR (Long et al. 2005). Although the effect was said not to be due to increased loading of Rheb with GTP (Long et al. 2005), other data indicate that amino acids did in fact promote the loading of Rheb with GTP (Smith et al. 2005; Roccio et al. 2006; Tzatsos and Kandror 2006; Sun and Chen 2008), but the reason for this increase in GTP loading was not explained. It is important to stress, however, that in TSC knockdown cells in which Rheb was fully charged with GTP, irrespective of the presence of amino acids, activation of MTOR remained amino acid dependent (Smith et al. 2005; Roccio et al. 2006). This indicated at least one other mechanism for amino acid activation of MTOR activity.

## Rag

A big step forward in the search for a mechanism of amino acid sensing was the demonstration that the Rag GTPases are essential for the activation of MTOR by amino acids (Kim et al. 2008; Sancak et al. 2008) and that active MTORC1 appeared to be localized at the lysosomal membrane (Sancak et al. 2010). The Rag proteins form heterodimers between RagA/B and RagC/D, and in its most active form, RagA/B is in the GTP form and RagC/D in the GDP form (Tsun et al. 2013). Interestingly, amino acids increased the charging of RagA/B with GTP (Sancak et al. 2008). It was proposed that the v-ATPase in the lysosomal membrane, in addition to its role in proton pumping, acts as the amino acid sensor in MTOR signaling (Zoncu et al. 2011). The v-ATPase responds to an increase in the intralysosomal, rather than the cytosolic, amino acid concentration with a conformational change. This causes increased binding of Ragulator, a scaffolding protein complex consisting of 5 different proteins with guanine nucleotide exchange activity toward RagA and RagB, which anchors the Rag proteins to the lysosomal surface (Bar-Peled et al. 2012). At the extralysosomal surface MTORC1 interacts with Rheb and becomes activated.

Recruitment of the MTOR–Rag protein complex to the lysosome requires binding to the tumor suppressor protein folliculin. Folliculin is a GTPase-activating protein that specifically regulates the nucleotide status of RagC/D, not of RagA/B. Folliculin binding to the lysosome further requires association with the folliculin interacting proteins-1 and 2 (Petit et al. 2013; Tsun et al. 2013). A protein complex, called GATOR, composed of two subcomplexes, GATOR-1 and -2, also interacts with the Rags. As folliculin, GATOR-1 acts as a GTPase-activating protein but in this case toward RagA and RagB, not RagC and D, and thereby suppresses MTORC1 activity (Bar-Peled et al. 2013). Inactivating mutations of its components, which may occur in cancer, makes MTORC1 signaling resistant to amino acid deprivation. GATOR-2, in turn, negatively regulates GATOR-1 so that inhibition of GATOR-2 suppresses MTORC1 signaling.

The v-ATPase-mediated mechanism of amino acid sensing by MTORC1 at the lysosomal surface requires the proton gradient across the lysosomal membrane (Settembre et al. 2012). The activity of the lysosomal proton-assisted amino acid transporter PAT1, responsible for the efflux of amino acids from the lysosomes, may control the concentration of amino acids within the lysosomal lumen and thus the extent of MTORC1 activation (Zoncu et al. 2011), but the participation of other amino acid transporters in the lysosomal membrane cannot be ruled out (Efeyan et al. 2012). If the mechanism is correct, it must be speculated that among the various amino acids leucine is specially active in inducing the conformation change of the v-ATPase. In vitro experiments with isolated lysosomes in which the conditions for binding to the lysosomal membrane, and activation, of MTORC1 were analyzed have suggested that this may, indeed, be the case (Zoncu et al. 2011).

The fact that MTOR activity in MTORC1 is determined by the size of the intralysosomal pool of amino acids implies that the use of compounds such as the v-ATPase inhibitor bafilomycin or the acidotropic agent chloroquine cannot be recommended to estimate autophagic flux by monitoring the accumulation of the autophagosomal marker LC3-II. This is because inhibition of proteolysis within the lysosomes will directly affect the intralysosomal pool of amino acids, and thus leads to underestimation of MTOR activity and overestimation of autophagic flux (Juhasz 2012; Klionsky et al. 2012).

Puzzling is a recent report showing that <10 % of total intracellular Rag colocalized with the lysosomes, although loss of Rag from the lysosomal fraction to the cytosol during cell disruption and fractionation by differential centrifugation on sucrose density gradients could not be excluded (Oshiro et al. 2013).

In addition to the localization of active MTOR, as part of MTORC1, at the lysosomal membrane, MTOR present in MTORC2 is localized at the mitochondria (Schieke et al. 2006; Ramanathan and Schreiber 2009; Betz et al. 2013), presumably at the mitochondrial-endoplasmic reticulum contact site where it controls mitochondrial function (Betz et al. 2013).

## Leucyl-tRNA synthetase

An early hypothesis proposed that free, uncharged, tRNA participates in amino acid sensing (Hara et al. 1998; Iiboshi et al. 1999). This hypothesis was based on observations with yeast in which, on amino acid starvation, free tRNA binds with high affinity to the protein kinase GCN2 (the equivalent of eIF2α-kinase) because the active center of GCN2 strongly resembles that of aminoacyl-tRNA synthetases (Hinnebusch 1997; Dong et al. 2000). GCN2 activation results in phosphorylation of eIF2α which then results in the derepression of GCN4 mRNA translation. GCN4 (equivalent to mammalian ATF4) is a transcriptional activator that promotes the transcription of many genes involved in nitrogen metabolism, not only genes involved in amino acid biosynthesis but also in autophagy (Natarajan et al. 2001; Tallóczy et al. 2002; B’chir et al. 2013). The particular potency of leucine in activating MTOR was proposed to be related to the frequency of utilization of this amino acid in protein synthesis and by the existence of multiple leucyl-tRNA synthetases arising from the sixfold codon degeneracy (Hara et al. 1998). Because uncharged tRNA did not affect the in vitro activity of immunoprecipitated MTOR, it was postulated that free tRNA interacted with an as yet unknown, signal-transduction component regulating MTOR (Iiboshi et al. 1999). Incompatible with the tRNA hypothesis, however, were kinetic considerations (the *K*_m_ of leucine for leu-tRNA synthetase being orders of magnitude lower than prevailing intracellular leucine concentrations) and because specific aminoalcohols, inhibiting leu-tRNA synthetase, did not affect MTOR signaling (Lynch et al. 2000) (contrast (Iiboshi et al. 1999)). Finally, at least in HEK-293 cells, free tRNA levels did appear to change by amino acid starvation (Dennis et al. 2001).

Interest in the tRNA mechanism revived with the finding that leucyl-tRNA synthetase directly binds to Rag GTPase in a leucine-dependent manner and functions as a GTPase-activating protein (GAP) for Rag GTPase to activate MTORC1 (Han et al. 2012). In this mechanism, leucylation of the tRNA is not required. It is sufficient that leucine binds to the leucine-binding domain of the leucyl-tRNA synthetase and activates the enzyme, as measured by ATP-[^32^P]PPi exchange activity (Han et al. 2012). In this context, it is noteworthy that diadenosine tetraphosphate (Ap4A), a byproduct of the aminoacyl-tRNA synthetase reaction, was previously proposed by us as a factor involved in MTOR stimulation by amino acids because Ap4A is a strong inhibitor of AMPK (Meijer 2008).

It is important to stress that the two mechanisms of amino acid sensing, discussed in the previous paragraphs, detect different pools of amino acids: The v-ATPase senses the intralysosomal pool of amino acids, while the leucyl-tRNA synthetase senses cytosolic leucine. It is perfectly possible that these two mechanisms coexist in amino acid sensing (Yoon et al. 2011; Duran and Hall 2012a).

## Glutamate dehydrogenase

Some years ago, on the basis of existing literature, we hypothesized that glutamate dehydrogenase (GDH), in addition to its role in amino acid catabolism, is involved in amino acid sensing and in controlling autophagy (Meijer 2008; Meijer and Codogno 2008, 2009). The arguments were as follows. As discussed earlier, this mitochondrial enzyme is specifically activated by leucine. In pancreatic β-cells, the ability of leucine (but not of valine or isoleucine) to stimulate production of insulin and to stimulate rapamycin-sensitive S6K phosphorylation was ascribed to stimulation of GDH (Xu et al. 2001). Moreover, a mutation in GDH, which results in overactivation of the enzyme, underlies the hyperinsulinism/hyperammonia (HHS) syndrome (Li et al. 2012b). A combination of glutamine (a glutamate donor) and leucine, which maximizes the flux through GDH, is most effective in stimulating MTOR and in inhibiting autophagic flux in several cell types, including a.o., β-cells and hepatocytes ((Meijer and Codogno 2009), for literature), as discussed earlier (cf. section “Discovery of amino acid signaling and its relationship with autophagy”). Recent studies by Duran et al. (2012) using both genetic and pharmacological methods have now provided strong experimental evidence that in the course of glutamine metabolism, GDH does, indeed, play a crucial role in the activation of MTOR. It was also demonstrated that it is the production of 2-oxoglutarate by GDH which stimulates loading of RagB with GTP. The link between 2-oxoglutarate and MTORC1 was proposed to be prolylhydroxylase which, in a HIF-1α-independent manner, somehow results in increased RagB^GTP^ (Duran et al. 2013). In these studies, the possibility of increased production of GTP by succinyl CoA synthetase in the course of 2-oxoglutarate oxidation in the mitochondria, suggested by us in the past (Meijer and Codogno 2009), was not explored.

By contrast, other data have indicated that the charging of RagA/B with GTP does not play a role in the mechanism of amino acid sensing. Thus, Rag heterodimers extracted from [^32^P]P_i_-labeled whole cells or from the pool associated with the lysosomal membrane exhibited constitutive [^32^P]GTP charging that was unaltered by amino acid withdrawal. In addition, in cells with mutant Rag which was unable to bind GTP, activation of MTOR by amino acids still occurred (Oshiro et al. 2013). Another concern is that dimethyl-2-oxoglutarate, a cell-permeable analog of 2-oxoglutarate, has been shown to inhibit, rather than stimulate, MTORC1 activity (Tan and Hagen 2013). The reason for these differences in results is not clear.

The importance of GDH in the activation of MTOR was also indicated, albeit indirectly, by studies of van der Vos et al. (2012) showing that overexpression of glutamine synthetase inhibited MTOR activity, inhibited the translocation of MTOR to the lysosomes and activated autophagy. Surprisingly, it was concluded that glutamine itself functions as an inhibitor of MTOR, and thus as an activator of autophagy, a conclusion which is in contrast to existing literature (see above). However, the fact that increased flux through glutamine synthetase results in increased flux through GDH, in this case in the direction of amination, i.e., from 2-oxoglutarate to glutamate, because of the use of glutamate for glutamine synthesis, was overlooked (Duran and Hall 2012b). In agreement with this interpretation is the finding that in glutamine-depleted cells pharmacological inhibition of glutamine synthetase greatly stimulated MTOR activity (Tardito et al. 2012).

Apart from a role of GDH in the production of 2-oxoglutarate for the activation of MTOR, it is also possible that NADPH, another product of the deamination reaction, activates MTOR, and inhibits autophagy, by eliminating reactive oxygen species (ROS) which are predominantly produced in the mitochondria (Meijer and Codogno 2009). This may occur by, e.g., the glutathione–glutathione reductase system, and/or through direct prevention by NADPH of ROS production at the FMN-a site of complex I of the mitochondrial respiratory chain (Albracht et al. 2011). In this context, it is worthwhile to note that glutaminase-2 (liver-type), in contrast to glutaminase-1 (kidney-type), is not sensitive to product inhibition (Kovacevic and McGivan 1983; Mates et al. 2013). This allows a high flux through glutaminase and GDH at a relatively high steady-state intramitochondrial glutamate concentration. This is not only of importance for production of NADPH via GDH with its high *K*_m_ for glutamate but it is also favorable for the synthesis of glutathione. Interestingly, not only does a high flux through GDH stimulate MTORC1 but, conversely, does MTORC1 activate GDH through transcriptional repression of SIRT4, the mitochondrial localized sirtuin that inhibits GDH (Csibi et al. 2013). This is very efficient from the point of view of metabolic regulation.

In addition to NADPH, 2-oxoglutarate, the other product of the GDH reaction, can also act as a scavenger of ROS, which oxidizes 2-oxoglutarate to succinate non-enzymically (Mailloux et al. 2007).

When the production of ROS exceeds its degradation, excessive ROS levels induce oxidative stress and damage of cellular components including DNA, proteins and lipids. A rise in ROS levels (e.g., in starvation) activates autophagy as a protective mechanism, and this occurs in a manner that is sensitive to antioxidants (Scherz-Shouval et al. 2007; Li et al. 2012a; Morales et al. 2014; Rahman et al. 2014) which, simultaneously, stimulate MTOR activity (Li et al. 2013). The importance of ROS in the initiation of autophagy was also indicated by the observation that knockdown of the antioxidant transcription factor Nrf2 in breast cancer cells substantially increased autophagy in response to oxidative stress (Rao et al. 2010). Possible targets of ROS contributing to the stimulation of autophagy by ROS are ATG4 (Scherz-Shouval and Elazar 2011), PKB (Rahman et al. 2014), Beclin1 (Bolisetty and Jaimes 2013) and AMPK (Toyoda et al. 2004).

Experiments very recently carried out in our laboratories (Lorin et al. 2013b) showed that the production of ROS by starved HeLa cells, a cervix cancer cell line, was suppressed by either a complete mixture of all amino acids or by a combination of glutamine plus leucine alone. Knockdown of GDH prevented these effects. At the same time, knockdown of GDH stimulated autophagy and inhibited MTOR signaling in the presence of amino acids. By what mechanism oxidative stress inhibits MTOR is unknown. Possibilities are increased AMPK and decreased PKB activities (cf. the previous paragraph). It is also possible that one, or more, of the components involved in the amino acid sensing mechanisms discussed above is redox sensitive. A very recent development is the finding that TSC1/TSC2 is a target for ROS, in this case of ROS produced by peroxisomes. Interestingly, both TSC1/TSC2 and Rheb were found to be bound to peroxisomes and TSC1/TSC2 became activated by ROS produced by the peroxisomes. This resulted in the conversion of peroxisome-bound Rheb^GTP^ to Rheb^GDP^ and in inhibition of lysosome-bound MTORC1 (Zhang et al. 2013). This was accompanied by activation of autophagy. How peroxisomal activation of TSC1/TSC2 and inhibition of Rheb caused inactivation of lysosomal MTORC1 was not clear because MTORC1 did not colocalize with the peroxisomes ((Benjamin and Hall 2013; Betz and Hall 2013), for discussion). An attractive mechanism would be that MTORC1, TSC1/TSC2 and/or Rheb shuttle between various sites in the cell where ROS is produced locally to bring the signal to the lysosomes which is considered to be the site responsible for amino acid activation of MTORC1. In line with this is, the very recent finding that deprivation of insulin and of amino acids recruits the TSC complex to the lysosomes where it acts to inactivate Rheb (Menon et al. 2014; Demetriades et al. 2014). It will be of interest to see whether a system similar to that found for peroxisomal ROS is also present for ROS produced by the mitochondria. Indeed, association of Rheb and MTORC1 with mitochondria cannot be excluded (Schieke et al. 2006; Groenewoud and Zwartkruis 2013).

In this context, it is important to note that glutamine deprivation in pancreatic cancer cells also resulted in increased ROS production (Son et al. 2013). Suppression of ROS by glutamine was ascribed to production of oxaloacetate from glutamine-derived aspartate, followed by its reduction to malate and subsequent production of NADPH (and pyruvate) by malic enzyme in the cytosol. Elimination of cytosolic aspartate aminotransferase, indeed, greatly increased production of ROS, which could be counteracted, at least in part, by addition of oxaloacetate (Son et al. 2013). However, net production of oxaloacetate from glutamine requires net production 2-oxoglutarate, and this cannot proceed without the participation of GDH or of glutamate pyruvate transaminase (Yang et al. 2009). The finding that addition of oxaloacetate in the absence of aspartate aminotransferase could suppress ROS accumulation is not surprising because α-oxoacids react non-enzymically with ROS (Holleman 1904; Andrae et al. 1985; Mailloux et al. 2007).

In retrospect, the participation of glutamate dehydrogenase in the control of autophagy is in agreement with the observation that autophagy was stimulated pharmacologically by the green tea component epigallocatechin gallate (Li et al. 2011; Zhou et al. 2014). This compound is a powerful inhibitor of glutamate dehydrogenase (Li et al. 2006), but the link between these phenomena was not made.

Involvement of GDH in amino acid sensing has serious consequences for the use of chloroquine in the measurement of autophagic flux. This compound not only increases the intralysosomal pH and in this way affects the intralysosomal amino acid pool, as discussed above (cf. section “Rag”) but, in addition, is also a potent inhibitor of GDH (Jarzyna et al. 1997). This, too, leads to overestimation of autophagic flux. Along the same line, stimulation of autophagy by ammonia (Eng et al. 2010; Harder et al. 2014) may be ascribed to the fact that this metabolite drives the GDH reaction in the direction of glutamate synthesis. An alternative, or perhaps additional, mechanism is activation of AMPK by ammonia (Harder et al. 2014). It must be pointed out that in the latter experiments, ammonia was used at mM concentrations. Although the data indicated that ammonia increased the rate of autophagosome formation, it is highly likely that under these conditions the intralysosomal pH was also affected. Autophagic flux under turnover conditions, as measured by 3-methyladenine-sensitive proteolysis, was not analyzed, however.

## PIK3C3 and p62/SQSTM1

Some years ago it was proposed that PIK3C3 is required for amino acid signaling, an effect that is mediated by an amino acid-induced rise in cytosolic Ca^++^, which results in increased binding of Ca^++^/calmodulin to, and activation of, PIK3C3 (Nobukuni et al. 2005; Gulati et al. 2008). Apart from the fact that a rise in Ca^++^/calmodulin stimulates autophagy through activation of AMPK by calmodulin-dependent kinase–kinase-β (Hoyer-Hansen et al. 2007; Meijer and Codogno 2009; Pfisterer et al. 2011; Ghislat et al. 2012) and that it is more likely that cytosolic Ca^++^ decreases rather than increases in the presence of amino acids (Meijer and Codogno 2009; Ghislat et al. 2012), although this issue has been controversial (Wauson et al. 2012), these observations were puzzling because PIK3C3 also participates in the formation of autophagosomes.

A similar problem relates to the proposal that p62/SQSTM1 participates in amino acid signaling (Duran et al. 2011), because this protein does not inhibit autophagy but rather is required for this process.

In order to solve the problem with PIK3C3, it was proposed that the enzyme is part of different protein complexes with different functions (Kim and Guan 2011; Ktistakis et al. 2012). Recent experiments indicate that this is, indeed, the case and that different PIK3C3 complexes exist which are differentially regulated by amino acids (Yuan et al. 2013; Kim et al. 2013a).

It has been proposed that amino acids, by locally activating the formation of PI(3)P, actually stimulate the production of PI(3,5)P_2_ by phosphatidylinositol-3-phosphate-5-kinase. PI(3,5)P_2_ is able to bind to RAPTOR where it serves to recruit MTOR downstream targets and/or to bring MTORC1 to the proper location in the cell (Bridges et al. 2012; Jin et al. 2014).

p62/SQSTM1 may also be compartmented because only a small part of the total cellular p62 is bound to the MTORC1 complex through its association with the Rag GTPases (Duran et al. 2011). As an alternative explanation, however, we propose that autophagy itself produces amino acids which then stimulate MTOR activity (Shigemitsu et al. 1999a; Beugnet et al. 2003; Yu et al. 2010; Inoki et al. 2012). Thus, overexpression of PIK3C3 or p62 initially activates autophagy, resulting in increased production of amino acids from proteins within the lysosomes. This increases the size of the intralysosomal pool of amino acids which is sensed by the v-ATPase in the lysosomal membrane and activates MTOR according to the mechanism discussed above. Conversely, if PIK3C3 or p62 becomes inhibited, autophagic flux declines, the intralysosomal amino acid pool decreases and MTOR becomes inhibited. The observation that in skeletal muscle of mice deficient of myotubularin, the lipid phosphatase responsible for the degradation of PI(3)P, autophagy is defective and MTOR overactivated (Fetalvero et al. 2013) may be explained similarly. Perhaps initially, autophagy is overactivated because of the rise in PI(3)P. This results in increased autophagic proteolysis, a rise in the concentration of lysosomal amino acids, upon which MTOR becomes activated and autophagy inhibited again.

## Phospholipase D

The phosphatidic acid-producing enzyme phospholipase D (PLD) (Fang et al. 2001) has also been implicated as one of the components taking part in the mechanism by which amino acids activate MTORC1.

It has been reported that amino acids activate PLD1 in a PIK3C3-dependent manner and that PLD1 is indispensable for translocation of MTORC1 to the lysosomes and its activation by amino acids (Yoon et al. 2011; Xu et al. 2011). In the absence of PIK3C3, addition of phosphatidic acid only activated MTORC1 when amino acids were also present. Addition of exogenous PI(3)P stimulated PLD1 activity in the absence of amino acids but did not activate MTORC1 (Yoon et al. 2011). These and other observations led to the suggestion that PLD1 may be part of the protein complex anchoring MTORC1 to the lysosomal membrane and that the PIK3C3-PLD1 pathway acts in parallel to the Rag pathway in regulating amino acid activation of MTORC1 (Yoon et al. 2011; Wiczer and Thomas 2012). Unexpectedly, the effect of PLD1 on autophagy has been controversial: both stimulation (Dall’Armi et al. 2010) and inhibition (Jang et al. 2014) of autophagy have been reported. It was suggested that this dual effect of PLD on autophagy, as with PIK3C3, is dependent on the subcellular localization of PLD (Jang et al. 2014). However, as discussed in the previous section, it cannot be excluded that the PIK3C3-phosphatidic acid pathway is actually required for autophagy but that MTORC1 becomes activated after the intralysosomal pool of amino acids has sufficiently expanded by autophagic proteolysis.

## Inositol polyphosphate multikinase

Inositol polyphosphate multikinase is another component implicated in the mechanism responsible for stimulation of MTOR activity by amino acids (Kim et al. 2011b). Independent of its catalytic activity the enzyme appeared to stabilize the binding between MTOR and RAPTOR in the MTORC1 complex through its aminoterminal amino acid sequence which forms a unique mammalian MTOR binding site (Kim et al. 2011b).

## MAP4K3

MAP4K3 is another protein that acts upstream (Findlay et al. 2007; Yan et al. 2010) of MTOR which becomes activated by amino acids. Its role in amino acid sensing is not entirely clear. It is unlikely that this kinase participates in either the v-ATPase or the leucyl-tRNA synthetase mechanism of amino acid sensing. Presumably, MAP4K3 is part of another pathway leading to amino acid-induced MTOR activation (Kim and Guan 2011).

## Plasma membrane amino acid receptor

Early evidence indicated that the amino acid receptor was intracellular rather than extracellular (Martin and Sutherland 2001; Christie et al. 2002; Beugnet et al. 2003). However, this may not entirely be true. There are indications that the plasma membrane can also contribute to amino acid sensing. Thus, it has been suggested that the plasma membrane of hepatocytes contains a leucine-specific receptor protein (which does not transport leucine) which controls autophagy independently of MTOR (Kanazawa et al. 2004).

In analogy with yeast, plasma membrane amino acid transporters have also been implicated in the sensing of (extracellular) amino acid availability by mammalian cells (Hundal and Taylor 2009; Kim and Guan 2011). Such a role was attributed to a transport protein that mediates the exchange between extracellular leucine and intracellular glutamine, which allows leucine to be transported against a concentration gradient (Nicklin et al. 2009). It must be pointed out, however, that an intracellular localization of the amino acid sensor implies that any process affecting the intracellular concentration of leucine, whether it is its transport across the plasma membrane or the rate of the intracellular metabolism of leucine, will affect the ability of leucine to stimulate MTOR activity.

Very recently, it was reported that the G-protein-coupled taste receptor complex T1R1/T1R3, an amino acid receptor in the plasma membrane, originally discovered in gustatory neurons as a detector of the umami flavor and present in many tissues, is an early sensor of extracellular amino acid availability (Wauson et al. 2012). Reduced expression of T1R1/T1R3 impaired the activation of MTOR by amino acids, caused mislocalization of MTORC1, and accelerated autophagy under nutrient-rich conditions. Interestingly, the intracellular concentration of amino acids, leucine included, was not affected by knockdown of the taste receptor even though the expression of several plasma membrane amino acid transporters greatly increased under these conditions (Wauson et al. 2012). The question of how inactivation of MTOR and activation of autophagy in T1R1/T1R3 receptor knockdown cells could occur in the absence of changes in intracellular amino acid concentrations remained unanswered. An obvious explanation could be that GDH was downregulated after knockdown of the taste receptor. But this is unlikely because this enzyme plays a central role in amino acid catabolism and its downregulation would have resulted in increased intracellular amino acid concentrations under these conditions, which was not observed. Another, plausible, possibility is that AMPK was activated. However, AMPK was inhibited, instead (Wauson et al. 2012). A third possibility, i.e., that leucyl-tRNA synthetase was affected by T1R1/T1R3 receptor knockdown, was not explored.

## Amino acid sensing and the concentration of cytosolic acetyl CoA

A very recent development has been the finding that the acetyltransferase EP300, responsible for the inhibitory acetylation of several ATG proteins, because of its low affinity (high *K*_m_) for acetyl CoA, acts as a sensor of cytosolic acetyl CoA which translates increases in cytosolic acetyl CoA into inhibition of autophagy (Mariño et al. 2014b). Thus, several experimental manipulations designed to alter cytosolic acetyl CoA, both in cultured cells and in vivo in mice, resulted in predicted changes in autophagic flux. Because the level of cytosolic acetyl CoA also strongly correlated with the activity of MTORC1 the possibility that the acetylation-dependent control of autophagy was indirect and mediated by MTORC1 could not be excluded (Mariño et al. 2014b).

In view of these fascinating observations, it is possible that amino acid catabolism may actually result in increased cytosolic acetyl CoA and in this way inhibits autophagy and stimulates MTORC1 signaling. This, then, would yet be another mechanism by which amino acids affect these pathways. Although this remains to be demonstrated, glutamate dehydrogenase, by stimulating synthesis of citrate followed by exit of citrate from the mitochondria and its cleavage by ATP-citrate lyase in the cytosol, can be expected to play an important role here, too.

## Amino acid signaling and autophagy: summary

In summary, on the basis of current literature, it is clear that there is not one unique mechanism of amino acid sensing. Rather, several mechanisms of amino acid sensing, leading to activation of MTOR, can operate. Whether these mechanisms are context- and/or cell type-dependent, or act in parallel, is currently not known.

Although amino acids can inhibit autophagy by activation of MTOR, they can also do so in an MTOR-independent manner. Thus, as we have seen, amino acids can decrease Beclin1-associated PIK3C3 activity because they promote the association between Beclin 1 and Bcl-2 through inhibition of JNK1-mediated phosphorylation of Bcl-2. In addition, amino acids may decrease AMPK activity which results in dephosphorylation, and inhibition, of Beclin1 and ULK1. This may explain why the inhibition of autophagy by amino acids is not always fully reversed by rapamycin. The inhibition of autophagy by insulin through protein kinase B-induced phosphorylation of Beclin1 and the transcription factor FoxO3 and through the direct inhibitory effects of PI(3,4)P2 and PI(3,4,5)P3 is also MTORC1 independent, and thus rapamycin insensitive.

## Conclusions

Our original discoveries of amino acid-stimulated, MTOR-mediated, signaling and its role in the control of autophagy, the regulation of these two pathways by the energy sensor AMPK and the role of phosphatidylinositol 3-phosphate in autophagy have opened new perspectives in the understanding of the regulation of cell metabolism. Given the enormous impact of autophagy on cell function, and its regulation by amino acid signaling, it is not surprising that research in these fields has expanded exponentially over the last two decades (Klionsky 2007; Ohsumi 2014). Proper in vivo manipulation of autophagy, either pharmacologically or by dietary restriction, under many pathological conditions may be used to the benefit of patients (Hermans et al. 2013). The safest way is dietary restriction. Although it is known for a very long time to be beneficial for health, e.g., in obesity, type 2 diabetes and aging, the underlying mechanisms are still not clear. A simple explanation is that dietary restriction acts as a two-sided sword. On the one hand, it reduces the redox pressure on the mitochondrial respiratory chain, and thus diminishes overproduction of potentially harmful ROS. On the other hand, dietary restriction also stimulates autophagy and thereby contributes to the improvement of cell function (Cavallini et al. 2008; Blagosklonny 2010; Gelino and Hansen 2012; Eisenberg et al. 2014).

## Abbreviations

4E-BP1: Eukaryotic translation initiation factor 4E binding protein 1
AA: Amino acids
AMBRA1: Activating molecule in Beclin1-regulated autophagy protein 1
AMPK: AMP-activated protein kinase
ATF4: Activating transcription factor 4
ATG: Autophagy related
BAD: Bcl-2-associated death promoter
BARKOR: Beclin1-associated autophagy-related key regulator
Bcl-2: B cell lymphoma 2
Bcl-xL: B cell lymphoma extra large
Beclin1: Bcl-2-interacting coiled-coil protein 1
BH3: Bcl-2 homology domain
BNIP3: Bcl-2/adenovirus E1B 19 kDa interacting protein 3
CHO: Chinese hamster ovary
DAPK: Death-associated protein kinase
DEPTOR: DEP domain-containing MTOR-interacting protein
DFCP1: Double FYVE-domain-containing protein 1
eEF2 kinase: Eukaryotic translation elongation factor 2 kinase
eIF2α kinase: Eukaryotic initiation factor 2α kinase
EP300: E1A binding protein p300
E1A: Adenovirus early region 1A
FIP200: Focal adhesion kinase family-interacting protein of 200 kDa
FoxO3: Forkhead box O3
GAP: GTPase-activating protein
GATOR: GTPase-activating protein toward Rags
Gcn2: General control non-depressible 2
Gcn4: General control non-depressible 4
GDH: Glutamate dehydrogenase
GEF: Guanine nucleotide exchange factor
HEK293: Human embryonic kidney 293
HIF-1α: Hypoxia-inducible factor 1α
IPMK: Inositol polyphosphate multikinase
IRS: Insulin receptor substrate
Lamp2: Lysosomal-associated membrane protein 2
LC3-I: Microtubule-associated protein 1A/1B-light chain 3
LC3-II: Microtubule-associated protein 1A/1B-light chain 3 conjugated with phosphatidylethanolamine
LRS: Leucyl-tRNA synthetase
MAP4K3: Mitogen-activated protein kinase kinase kinase kinase 3
mLST8: Mammalian lethal with sec-13 protein 8 (also GβL)
mSin1: Mammalian stress-activated MAP kinase-interacting protein 1
MTOR: Mechanistic target of rapamycin (formerly mTOR, mammalian target of rapamycin)
MTORC: Mechanistic target of rapamycin complex
Nrf2: NF-E2-related factor 2
PDK1: Phosphoinositide-dependent kinase 1
PE: Phosphatidylethanolamine
P_i_: Inorganic phosphate
PI(3)P: Phosphatidylinositol 3-phosphate
PI(3,4)P2: Phosphatidylinositol 3,4-biphosphate
PI(3,4,5)P3: Phosphatidylinositol 3,4,5-triphosphate
PIK3C1: Phosphatidylinositol 3-kinase class I
PIK3C3: Phosphatidylinositol 3-kinase class III
PIK3R4: Phosphatidylinositol 3-kinase regulatory subunit 4; also hVps15, formerly called p150
PKB: Protein kinase B (formerly AKT)
PLD: Phospholipase D
PRAS40: Proline-rich AKT substrate 40
PROTOR: Protein observed with RICTOR
PTEN: Phosphatase and tensin homolog deleted on chromosome 10
Rag: Ras-related GTP-binding protein
RAPTOR: Regulatory-associated protein of MTOR
Rheb: Ras homolog enriched in brain
RICTOR: Rapamycin-insensitive companion of TOR
ROS: Reactive oxygen species
S6: Ribosomal protein S6
S6K: 70 kDa S6 kinase (formerly p70S6K)
SQSTM1: Sequestosome 1
T1R1: Taste receptor type 1 member 1
T1R3: Taste receptor type 1 member 3
TFEB: Transcription factor EB
TSC: Tuberous sclerosis
ULK1/2: Unc-51-like kinase1/2
UVRAG: UV irradiation resistance-associated gene
v-ATPase: Vacuolar ATPase
VMP1: Vacuole membrane protein 1
Vps15: Vacuolar protein sorting 15
Vps34: Vacuolar protein sorting 34
WIPI-1/2: WD repeat domain phosphoinositide-interacting protein 1/2
